# Seasonal cycles, phylogenetic assembly, and functional diversity of orchid bee communities

**DOI:** 10.1002/ece3.1466

**Published:** 2015-04-13

**Authors:** Santiago R Ramírez, Carlos Hernández, Andres Link, Margarita M López-Uribe

**Affiliations:** 1 University of California DavisOne Shields Ave, Davis, California, 95616; 2 Departamento de Ecología, Facultad de Estudios Ambientales y Rurales, Pontificia Universidad JaverianaBogotá, Colombia; 3 Departamento de Ciencias Biológicas, Universidad de Los AndesBogotá, Colombia; 4 Department of Entomology, North Carolina State UniversityRaleigh, North Carolina, 27695

**Keywords:** Biodiversity patterns, euglossini, orchid bees, pollinators

## Abstract

Neotropical rainforests sustain some of the most diverse terrestrial communities on Earth. Euglossine (or orchid) bees are a diverse lineage of insect pollinators distributed throughout the American tropics, where they provide pollination services to a staggering diversity of flowering plant taxa. Elucidating the seasonal patterns of phylogenetic assembly and functional trait diversity of bee communities can shed new light into the mechanisms that govern the assembly of bee pollinator communities and the potential effects of declining bee populations. Male euglossine bees collect, store, and accumulate odoriferous compounds (perfumes) to subsequently use during courtship display. Thus, synthetic chemical baits can be used to attract and monitor euglossine bee populations. We conducted monthly censuses of orchid bees in three sites in the Magdalena valley of Colombia – a region where Central and South American biotas converge – to investigate the structure, diversity, and assembly of euglossine bee communities through time in relation to seasonal climatic cycles. In particular, we tested the hypothesis that phylogenetic community structure and functional trait diversity changed in response to seasonal rainfall fluctuations. All communities exhibited strong to moderate phylogenetic clustering throughout the year, with few pronounced bursts of phylogenetic overdispersion that coincided with the transition from wet-to-dry seasons. Despite the heterogeneous distribution of functional traits (e.g., body size, body mass, and proboscis length) and the observed seasonal fluctuations in phylogenetic diversity, we found that functional trait diversity, evenness, and divergence remained constant during all seasons in all communities. However, similar to the pattern observed with phylogenetic diversity, functional trait richness fluctuated markedly with rainfall in all sites. These results emphasize the importance of considering seasonal fluctuations in community assembly and provide a glimpse to the potential effects that climatic alterations may have on both pollinator communities and the ecosystem services they provide.

## Introduction

Communities are made of populations of multiple lineages that coexist in time and space, and understanding the mechanisms that control their diversity and structure is a fundamental goal of ecology and evolution. A variety of biotic and abiotic factors may influence the process of community assembly, including – but not exclusively – competition, habitat filtering, species pool diversity, and dispersal capacity (Chase 2003). Over the past decade, a variety of novel methods have been developed to incorporate phylogenetic information of coexisting lineages to investigate the mechanisms that control community structure, diversity, and assembly (Webb 2000; Webb et al. 2002; Cavender-Bares et al. 2009; Swenson 2013). Phylogenetic information may reveal the relative contributions of competitive exclusion and habitat filtering to community assembly. For example, a community may be composed of mostly unrelated lineages (phylogenetic overdispersion) if competitive exclusion is stronger between related taxa due to their ecological similarity (Cavender-Bares et al. 2009). Conversely, a community may be composed of mostly related lineages (phylogenetic clustering) if environmental factors filter colonization by lineages exhibiting phylogenetically conserved traits (Webb 2000; Webb et al. 2002). Although the use of phylogenetic methods to investigate the process of community assembly has become popular in recent years, competitive exclusion may not always be strong among related lineages (Venail et al. 2014), and thus, competition can produce opposing patterns of phylogenetic community assembly (Mayfield and Levine 2010). Hence, more comprehensive approaches that incorporate both phylogenetic information and functional trait data are desirable and may be better suited to infer the mechanisms governing community assembly (Laliberté and Legendre 2010; Swenson 2013).

Bees provide crucial pollination services to both agricultural (Klein et al. 2007; Brown and Paxton 2009) and natural ecosystems (Willmer 2011). Recent studies have raised concerns about sharp population declines of both native and managed bee populations, suggesting the possibility of a global pollination crisis (Potts et al. 2010; Cameron et al. 2011; Bartomeus et al. 2013). Although the precise causes behind widespread bee declines remain unknown, likely contributing factors include habitat degradation, pervasive pesticide use, and pathogen spillover from managed bee populations (Cox-Foster et al. 2007; van Engelsdorp et al. 2009; Dainat et al. 2012; Fürst et al. 2014). Bee communities in tropical regions are highly diverse, and although little is known about their population dynamics, some species are experiencing pronounced population declines (Roubik 2001; Villanueva-G et al. 2005). Moreover, in addition to having low population densities, many tropical plant taxa strongly depend on bee pollinators for cross-fertilization (Vamosi et al. 2006), and therefore, declining tropical bee populations could potentially lead to pervasive pollen limitation and low reproductive rates in a wide range of plant taxa (Burd 1994; Vamosi et al. 2006). Our limited knowledge on how tropical bee communities respond to biotic and environmental conditions has impeded a clear understanding of the potential effects that habitat disturbance, forest fragmentation, and climatic alteration may have on bee-mediated pollination services (Klein et al. 2007; Brown and Paxton 2009).

The tribe Euglossini (Hymenoptera: Apidae) includes approximately 240 species grouped in five genera (*Euglossa* Friese*, Eufriesea* Cockerell*, Eulaema* Lepeletier*, Exaerete* Hoffmannsegg*,* and *Aglae* Lepeletier and Serville) that are distributed from northern Mexico to northern Argentina (Ramírez et al. 2002, 2010). Euglossine bees comprise up to ∼25% of the local bee communities in lowland wet forests (Roubik and Hanson 2004), and their abundance is apparent in both pristine and disturbed habitats (López-Uribe et al. 2008; Brosi 2009). The extraordinary flight performance of euglossine bees (Dudley 1995; Darveau et al. 2005; Wikelski et al. 2010) make them ideal pollinators of scattered plant populations, particularly in fragmented forest patches (Janzen 1971). Unlike most insects, male euglossine bees acquire and accumulate perfume compounds from flowers and other sources, including hundreds of orchid species to subsequently use in courtship display (Williams 1982; Williams and Whitten 1983; Eltz et al. 1999; Ramirez et al. 2011). Displaying male bees expose perfume mixtures, presumably to convey species-specific recognition and/or mate choice signals to females (Zimmermann et al. 2009). The discovery of this behavior in the 1960s facilitated the study of euglossine bee communities with the use of artificial chemical baits (Vogel 1963; Dodson et al. 1969).

Multiple biotic and abiotic variables control the diversity, composition, and temporal fluctuations of insect communities. In particular, the assembly of bee pollinator communities is strongly influenced by climatic factors as well as variation in food resource availability (e.g., floral nectar and pollen), which in turn also fluctuate in response to climatic variables. A recent study found that orchid bee communities exhibit a hump-shaped pattern of phylogenetic diversity in the Amazon, with their diversity likely corresponding to historic climate fluctuations (Abrahamczyk et al. 2013). In addition, several studies have previously documented the influence of climatic variables in the assembly of euglossine bee communities at both temporal and spatial scales (Nemesio and Silveira 2007; Abrahamczyk et al. 2011a,b; Nemesio and Vasconcelos 2013).

**Figure 1:**
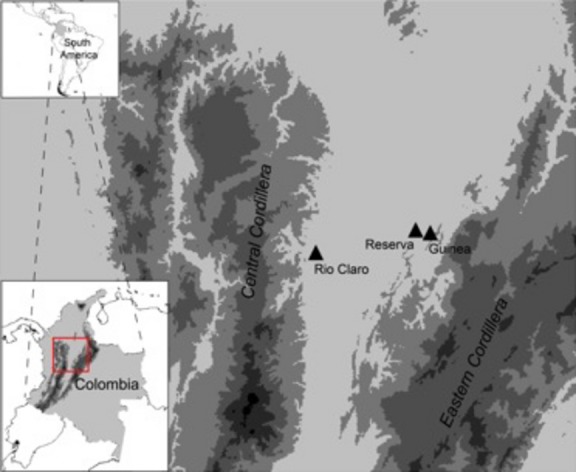
Geographic location of the three sampling locations in the Magdalena valley between the Eastern and Central Cordilleras of Colombia.

In this study, we conducted a yearlong census of euglossine bee communities in three different sites in the lowland rainforest of the Magdalena Valley of Colombia (Fig.1). We investigated the structure, diversity, and assembly of euglossine bee communities through time in relation to seasonal climatic variables. In particular, we tested the hypothesis that rainfall seasonal cycles mediate phenological fluctuations in phylogenetic and functional trait diversity of euglossine bee communities.

## Methods

### Study sites

We sampled communities of euglossine bees in three different tropical rainforest sites located between the Eastern and Central Cordilleras of the Magdalena Valley of Colombia (Fig.1). The Reserva el Paujil (hereafter Reserva) is located at 250 m above sea level (06°02′40.8″N 74°16′07.5″W). The Reserva site consists of approximately 80 hectares of forest fragments that were recently connected to a ∼1000 hectare forest. We conducted our survey in a forest that was selectively logged 10 years prior to the onset of this study. The surrounding area was logged and used for cattle ranching prior to becoming a private reserve in 2004, when the Fundación ProAves acquired the land for conservation (Aldana et al. 2008). The second site, Vereda La Guinea (hereafter Guinea) (06°01′18.1″N 74°11′55.1″W), is located at an elevation of 600 m in a large patch of ∼60,000 hectares of continuous forest. This area has been selectively logged for hardwood timber, but otherwise the area remains relatively undisturbed. The third site, Reserva El Cañon de Rio Claro (hereafter Rio Claro) (5°53′N 74°53′W), ranges in elevation between 300 and 600 m and is located at the southeastern part of the Magdalena valley, along the Central Cordillera, in the Colombian province of Antioquia. This reserve has been protected since 1976 and consists of ∼400 hectares of relatively undisturbed tropical rainforest that has experienced low-intensity timber extraction; the surrounding area has been severely disturbed for mining.

All three sites experience a bimodal climatic regime with two dry seasons (December–March and July–August), each followed by a wet season (May–June and September–November). The maximum daily temperatures in Reserva and Guinea are on average 32°C (range 26–37). The maximum relative humidity averages 90.5% ± 3.7 (range 78.0–98.0), and the minimum relative humidity averages 59.5% ± 8.0 (range 26.5–83.0). In Rio Claro, the average temperature is 26°C, and the average relative humidity is 79.4% (range 80–90) (IDEAM, Instituto de Hidrología, Metereología y Estudios Ambientales de Colombia).

### Sampling methods

In the Reserva and Guinea sites, male euglossine bees were sampled for a period of 11 months from June 2006 through May 2007 (except April 2007). In Rio Claro, sampling was conducted from October 2007 to September 2008. At each site, we sampled bees at a single location once every month for 5 h between 08:00 AM and 1:00 PM. We used five chemical baits: cineole (CIN), 1,4-dimethoxybenzene (DMB), methyl cinnamate (MC), methyl salicylate (MS), and vainillin (VAI) (Sigma-Aldrich, St. Louis, MO). We applied each chemical to a small square (5 × 5 cm) of absorbent paper clipped to a tree trunk at a height of 1.5 m.

Baits were placed along an existing trail 10 m away from each other, along mountain ridges. CIN and VAI were replenished every 1.5 h, and the remaining baits were replenished every 2.5 h. Censuses were conducted only during sunny days or slightly cloudy days to avoid short-term variability between samplings rounds. We sequentially collected bees that visited each of the five chemical baits using entomological nets and placed bees in 95% ethanol. Specimens were pinned and deposited at the Museo de Historia Natural of the Universidad de Los Andes (Bogota, Colombia). Species identification was carried out using reference collections of regional euglossine bees as well as taxonomic keys (Bonilla-Gómez and Nates-Parra 1992; Roubik and Hanson 2004).

### Phylogenetic analyses

To reconstruct a species-level phylogeny of the euglossine bee lineages present in our three communities, we used DNA sequence data generated in our previous study (Ramírez et al. 2010). Briefly, we used a total of ∼4.0 kb of DNA sequences from three nuclear and one mitochondrial loci; including 1200 base pairs of the mitochondrial cytochrome oxidase, *CO1*; 1200 base pairs of the nuclear elongation factor 1-alpha (*EF1-α*); 700 base pairs of the arginine kinase, *ArgK*; and 800 base pairs of the RNA polymerase II (*Pol-II*). We performed a new set of phylogenetic tree searches with the software package MRBAYES v3.2.2*,* where we assigned each locus a different model of sequence evolution. Tree searches were performed assuming multiple models of sequence evolution for each locus, and Markov Chain Monte Carlo (MCMC) searches were made for 10 million generations, sampling every 1000 generations, for a total of 10,000 trees. We estimated model parameters during runs and Bayesian posterior probabilities as the proportion of trees sampled; the trees obtained in the first 1 million generations were discarded. We determined models of sequence evolution with the software package Modeltest.

### Diversity analysis

We estimated species richness using the Jackknife estimator (to correct for sample size) and diversity using Shannon–Wiener (H’) and Simpson (D) indexes. In addition, we used nonmetric multidimensional scaling (MDS) to compare monthly measurements of community composition, as implemented in the R software package ecodist v1.2.2.

### Phylogenetic community assembly

We calculated two different metrics of phylogenetic community structure: phylogenetic diversity (PD) and mean pairwise phylogenetic distance (MPD). The phylogenetic diversity (PD) index is one of the earliest metrics developed to investigate relatedness within a community, and it measures the sum of the total branch lengths accumulated among the lineages present in a community (Faith 1992). The MPD index calculates the mean pairwise phylogenetic distance among all species in a community. Both indices can be corrected for communities of different sizes using the standardized effect size (SES) correction. This correction is calculated as the difference between the phylogenetic distances in the observed community and a set of null communities generated by random processes, divided by the standard deviation of the phylogenetic distances in the null communities (Kembel et al. 2010). We estimated SES-PD and SES-MPD to allow comparison among communities of different richness values. SES-PD and SES-MPD indices were estimated for each month in each community. SES-MPD is related to the previously used net relatedness index (NRI). This metric is a standardized measure of the mean pairwise phylogenetic distance of taxa in a sample, relative the phylogeny of the species pool. In fact, SES-MPD equals −1*NRI.

The species pool for the three communities combined consisted of a total of 50 taxa. Measures of phylogenetic and functional diversity are calculated relative to this source pool. Because the three communities we studied are located in the same biogeographic region, we assumed that the source pool is the same for all three locations. In fact, previous studies have documented the presence of these species in the region (Bonilla-Gómez and Nates-Parra 1992; Ramírez et al. 2002). In addition, we assume that the source pool does not change over time due to large-scale movement or migration events, as female euglossine bees are central place foragers (Roubik 1989). All the phylogenetic diversity analyses were performed using the software package Picante v1.6-1 (Kembel et al. 2010). To test whether abrupt changes in phylogenetic community structure coincide with shifts in rainfall patterns, we calculated a rainfall differential (*D*) as abs (*R*_t_ − *R*_t + 1_), where *R*_t_ is the average rainfall at time interval *t*. We used linear regression models to determine the relationship between *D* and SES-PD. Thus, a positive correlation between D and SES-PD would indicate that seasonality patterns of community assembly are correlated with changes in rainfall regimes.

### Functional morphological diversity

For each taxon in our 50 species pool, we measured the (1) body length, (2) body width (the distance between the tegulae at the wing base), (3) body mass (as estimated via dry weight), and (4) tongue length (length of the prementum). We measured and averaged a minimum of three individuals per species. We used the R software package FD v1.0-11 to compute different functional diversity metrics. We assembled a species-by-trait matrix, and in combination with the phenological data, we calculated functional evenness, functional diversity, functional richness, and functional disparity over time for each of the three communities. The software *FD* computes different multidimensional functional diversity indices. We used the *dbFD* function to calculate the indices indicated above for each month at each locality.

## Results

In total, we collected 4784 male euglossine bees of 50 species belonging to five genera in the tribe Euglossini (Table S1, Fig. S1). Overall, the most diverse and abundant genus was *Euglossa* (38 species, 4287 individuals) followed by *Eulaema* (5, 361), *Eufriesea* (4, 42), *Exaerete* (2, 90), and *Aglae* (1, 1). Total abundance of euglossine bees was highest at Reserva with 2013 individuals from 40 species, followed by Guinea with 1619 individuals from 41 species and Rio Claro with 1152 individuals from 34 species. In all three communities, nine species accounted for >60% of the total number of bees collected (Table S2). The most abundant species at Reserva and Rio Claro were *Euglossa imperialis* Cockerell and *Euglossa ignita* Smith; in contrast, *Euglossa mixta* Friese and *Euglossa cognata* Moure were the dominant species in the relatively undisturbed forests of Guinea. Overall, 33 species (67%) had a relative abundance >1% of the total individual bees collected (Fig. S3).

**Figure 2:**
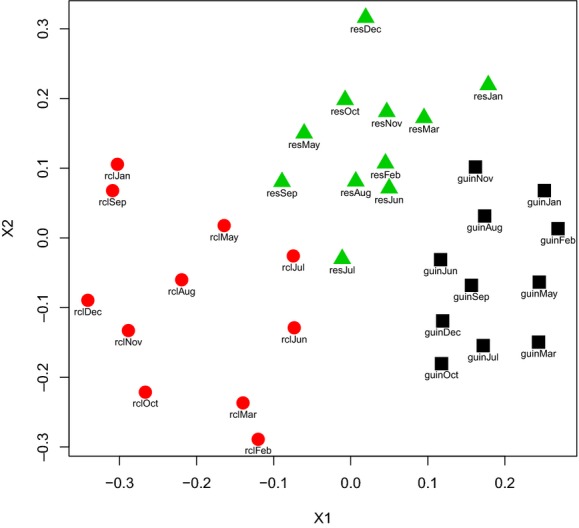
Nonmetric Multidimensional scaling (nMDS) plot demonstrating composition differences between three communities of euglossine bees sampled monthly (Guinea = guin; Rio Claro = rc; Reserva = res).

The average number of species per month was 21.9 ± 4.08 (median = 23, range 18–32) in Reserva, 26.2 ± 2.77 (median = 26, range 21–28) in Guinea, and 17.3 ± 2.61 (median = 17, range 14–22) in Rio Claro (Fig.2). Most of the bee species sampled showed modest seasonality (Fig.2). A total of 18 species (including the 16 most abundant), corresponding to 89% of all individuals, were collected every month. Furthermore, all of the 24 most abundant species (96% of the individuals) were collected for at least 8 months (Fig.2). In all sites, more than 90% of the species had been collected after 7 months of sampling, and by the 9th month, all species had been sampled in each community (Figs S2 and S3).

**Figure 3:**
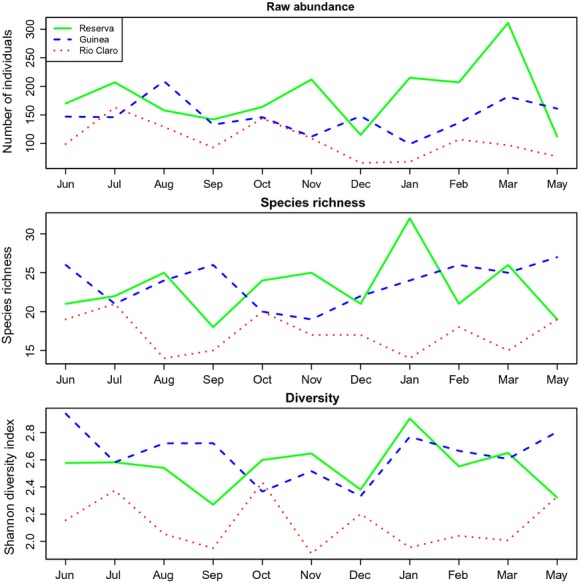
Total raw abundance values (top panel), species richness (middle panel), and diversity (bottom panel) of euglossine bees in three communities in the Magdalena Valley over the course of 11 months of sampling.

Jackknife estimator for species richness resulted in 43.63 (SE = 2.26), 42.63 (SE = 2.45) and 39.45 (SE = 2.82) in Reserva, Guinea and Rio Claro, respectively (Table S3). The Shannon diversity (H’) indices were lower at Rio Claro (H’ = 2.36) and Reserva (H’ = 2.78) than those of Guinea (H’ = 2.93) (Table S3). Despite the shared species and structure similarity among all three communities, our nonmetric multidimensional scaling analysis separated monthly censuses per community, with virtually no overlap among communities (Fig.3). A similarity percentage (SIMPER) analysis of all pairwise comparisons revealed that five species jointly explained >50% of the differentiation between communities: *Euglossa chlorina* Dressler, *E. cognata* Moure, *E. ignita* Smith, *E. imperialis* Cockerell, and *E. mixta* Friese (Table1).

**Table 1:** Similarity percentage (SIMPER) analysis indicating the relative contribution and cumulative contribution species abundances to the differences between communities

Community pairwise comparison	Species	Relative contribution (%)	Cumulative sum (%)
Reserva – Guinea	*Euglossa mixta*	5.66	11.16
	*Euglossa imperialis*	4.1	19.26
	*Euglossa ignita*	3.4	26.02
	*Eulaema meriana*	3.2	32.52
	*Euglossa cognata*	3.16	38.76
	*Eulaema cingulata*	3.14	44.97
	*Euglossa chlorina*	2.96	50.81
Reserva – Rio Claro	*Euglossa imperialis*	10.71	18.39
	*Eulaema meriana*	4.64	26.36
	*Euglossa chlorina*	4.53	34.15
	*Euglossa cognata*	4.19	41.36
	*Euglossa ignita*	3.54	47.45
	*Euglossa tridentata*	2.61	51.94
Guinea – Rio Claro	*Euglossa imperialis*	13.1	21.95
	*Euglossa cognata*	5.21	30.68
	*Euglossa chlorina*	4.59	38.38
	*Euglossa mixta*	3.7	44.59
	*Euglossa ignita*	3.7	50.8

Cineole attracted the highest number of bee species: 32 in Reserva and 30 in La Guinea (Fig. S4). DMB was also visited by a larger number of species (*N* = 32), and VAI was the most species-specific bait (*N* = 16). CIN also attracted the highest number of individuals (1631), followed by DMB (1404), MS (1126), MC (271), and VAI (254) (Fig. S4). We found significant differences in the number of species attracted by the chemical baits among the sampled sites (*χ*^2^ = 50.47, df = 11, *P* < 0.001) mainly explained by the high number of bees visiting DMB at Guinea. In fact, when bees attracted to this bait were removed from the analysis, the differentiation between sites decreased significantly (*χ*^2^ = 10.09, df = 9, *P* = 0.073).

**Figure 4:**
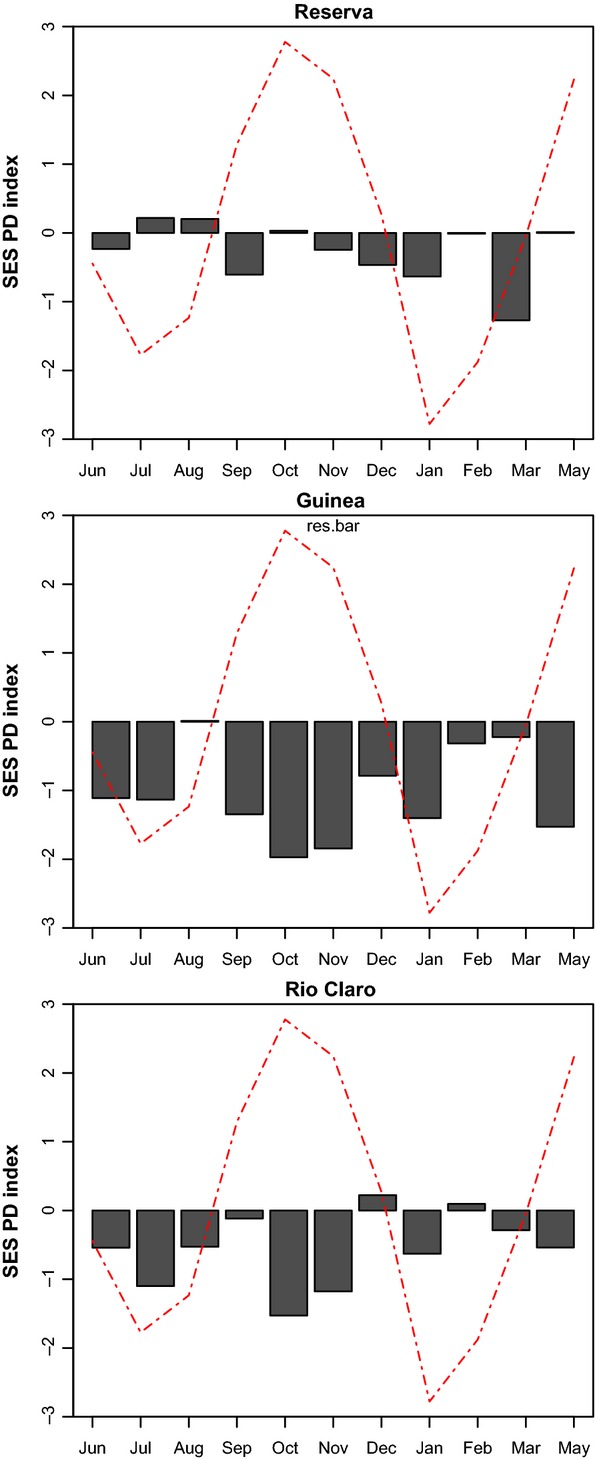
Estimated standardized phylogenetic diversity (SES-MPD) in three communities of euglossine bees in the Magdalena Valley, Colombia, over the course of 1 year. Average monthly rainfall patterns are superimposed for comparison (dotted line).

**Figure 5:**
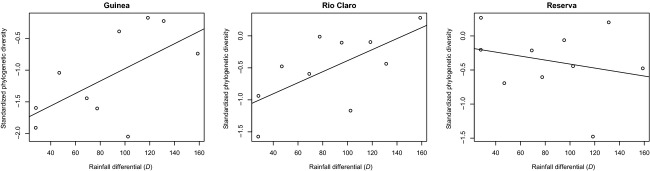
Linear regression models between standardized phylogenetic diversity and the rainfall differential (*D*). D is calculated as abs (*R*_t_ − *R*_t + 1_), where *R*_t_ is the average rainfall at time interval *t*. Linear regressions were positive and significant for Guinea (*P *=* *0.05) and Rio Claro (*P *=* *0.038), but not for Reserva (*P *=* *0.47).

The estimated indices of standardized phylogenetic diversity indicate that all three communities of euglossine bees exhibited strong to moderate phylogenetic clustering throughout the year (Fig.4). Negative SES-PD values (<−1) correspond to a pattern of phylogenetic clustering and positive values (>1) are congruent with a pattern of phylogenetic overdispersion in the community. Values close to zero indicate a random draw from the communities with respect to phylogeny. The SES-PD indices were positive for 8 months in La Reserva SES-PD with values ranging between 0 and 1; only 3 months had negative values (September, January, and March). In Guinea, all months (except August) exhibited negative SES-PD values (Fig.4). In Rio Claro, all months (except December and February) exhibited negative values of SES-PD. In the communities from Guinea and Rio Claro, there was a strong positive correlation between the rainfall differential *D* and the values of SES-PD, indicated by synchronous shifts in phylogenetic community assembly that coincide with the onset of the rainy season in August–September and the onset of the dry season in January–February (Fig.5). In Reserva, we did not find a correlation between D and SES-PD (Fig.5). Because both rainfall and community structure were measured in consecutive months throughout the year, there is the possibility of temporal autocorrelation in the dataset. Thus, we conducted a detrending of our time series using the R package pracma.

We measured functional diversity using a suite of morphological characters, including body size, body mass, and tongue length. These measurements, in conjunction with community data, revealed that functional diversity, functional evenness, and functional disparity remained constant throughout the year in all three communities (Figs S5–S9). Conversely, functional richness fluctuated substantially between our monthly censuses in all three communities and exhibited a similar pattern of temporal shifts to that observed for phylogenetic diversity indices (SES-MPD) with the largest shifts concentrated in the transition between rainy and dry seasons (Fig. S7). We calculated the phylogenetic signal statistic *K* for each of the traits included in our matrix, namely tongue length, body length, tegular distance, and body mass. All these character traits exhibit *K* values >1, indicating a strong phylogenetic signal and conservatism of traits (tongue length = 1.035, body length = 2.528, tegular distance 3.294, body mass = 4.468, Figs S10–S12).

## Discussion

Euglossine bees are one of the most abundant and diverse lineages of insect pollinators in lowland tropical America. Our yearlong survey of three communities in the Magdalena valley revealed one of the most species-rich orchid bee faunas from the neotropical region, surpassed only by communities from Panama (Roubik 2001, Table S5). Species richness and diversity were slightly higher throughout the year in the Reserva site, followed by Guinea, and both of these sites had considerably higher indices than the overall values observed in Rio Claro. These differences among communities likely correspond to a decreasing precipitation gradient that runs east to west in the Magdalena River Valley. In fact, several species of bees that are typically associated with wet forests (e.g.*, Eulaema bombiformis* Packard and *Euglossa intersecta* Latreille) were entirely absent in the Rio Claro site. The varying degrees of habitat quality and forest fragmentation among sites may also explain the observed differences between communities (Hedström et al. 2006; Nemesio and Silveira 2007). We also found that the relative ranked abundances of most species (Fig. S3) were equivalent among all sites, and the most abundant taxa in our species pool (e.g.*, Euglossa imperialis* Cockerell and *E. ignita* Smith) are also some of the most widespread and common species in the wet forests of Costa Rica, Panama, and Peru (Table S4). This observation is congruent with the hypothesis that the relative abundance of a species within a given community is correlated with its niche breadth, a phenomenon known as the abundance–range size relationship (Gaston et al. 1997).

Our study revealed pronounced seasonal and synchronous fluctuations in the structure of all three communities. These changes in community composition coincided with the transition from dry-to-wet and wet-to-dry seasons during the months of August–September and January–February, respectively. Several mechanisms may underlie these fluctuation patterns, and given their synchronicity in all sites, we hypothesize that the same mechanism(s) regulate population dynamics at all three sites. These fluctuations in community structure and population dynamics may be associated with floral resource availability (Heithaus 1979). In topical wet forests, flowering phenology is triggered by slight changes in light availability due to cloud cover and/or subtle variations in day length (Zimmerman et al. 2007). Like many wet forests of the neotropical region, the Magdalena Valley is characterized by having two wet seasons and two dry seasons, as caused by the latitudinal oscillations of the intertropical convergent zone. These seasonal transitions are known to induce flowering phenology in numerous plant taxa and the insect communities that feed on them, including bees (Heithaus 1979). Although the precise mechanisms that regulate the population dynamics of euglossine bees within a community remain unknown, likely candidates include emergence rates, mortality rates, and migratory movements. Although male euglossine bees are known to fly long distances (Janzen 1971; Wikelski et al. 2010), and therefore, some level of population fluctuations should be expected from migration alone, it is unlikely that the pronounced fluctuations that we observed were driven by massive migratory movements of male bees (Ackerman 1983). Hence, seasonality on brood cell construction, adult emergence, and/or mortality likely control the temporal fluctuations in community composition.

### Phylogenetic community assembly

Phylogenetic diversity fluctuated synchronously in all three communities, and all sites exhibited either negative or values close to zero of standardized mean phylogenetic diversity (SES-MPD) throughout most of the year, indicating moderate to strong phylogenetic clustering. The pronounced synchronous bursts of community change toward phylogenetic overdispersion coincided with the transition from wet-to-dry and dry-to-wet seasons. These fluctuations were correlated with changes in species richness and community diversity and were mainly driven by species in the genera *Eufriesea* and *Euglossa*. This temporal variation in the structure of euglossine bee communities may reveal the relative contributions of different ecological processes to the shaping of insect communities, including resource competition and habitat filtering. Equivalent to our results, previous studies with euglossine bee communities from the Amazon region have shown that phylogenetic diversity changes across latitudinal gradients, with decreasing phylogenetic diversity toward more seasonal climates (Abrahamczyk et al. 2011a, 2013). Thus, it is possible that the same ecological processes that drive phylogenetic diversity at a geographic scale shape communities at temporal scales within a given community. Previous research has focused on phylogenetic diversity between forest types, geographic clines, and climatic gradients (Graham et al. 2009; Gómez et al. 2010; Machac et al. 2010; Hoiss et al. 2012; Brehm et al. 2013), and thus, this study provides a first glimpse on the temporal changes in phylogenetic structure within the same community. However, to better understand the mechanisms that drive community assembly at both spatial and temporal scales, it is necessary to elucidate the link that exist between community dynamics, phylogenetic relatedness, and trait similarity.

### Functional diversity

Functional diversity is determined by the distribution of species abundances and richness in a community, and the relative contribution of individual taxa to the overall functional diversity in a community depends on the trait dimensionality (Petchey and Gaston 2002). For each community, we estimated functional trait diversity, richness, evenness, and disparity. Despite the observed pronounced heterogeneity in functional traits (body size and proboscis length) within and among communities and the seasonal variation in phylogenetic diversity, we found that functional trait diversity remained constant through time within each community. However, similar to the pattern observed with phylogenetic diversity, functional richness fluctuated markedly with rainfall in all sites. Temporal fluctuations in functional richness may directly impact the spectrum of pollination services available to plant communities at any given time. Thus, the diversity and relative abundance of functional traits of bee pollinators, rather than the actual taxa, are more likely to have an effect on the plant communities. Moreover, we detected a strong phylogenetic signal (*K*) in all the traits measured (body length, body width, body mass, and tongue length). This observation lends support to the idea that nonrandom fluctuation in community composition may translate into pronounced shifts in the diversity and relative abundance of functional traits, which could have cascading effects on pollination services. These results emphasize the importance of considering seasonal fluctuations in community assembly and provide a glimpse to the potential effects that climatic alterations may have on pollinator communities and the ecosystem services they provide.

### Biogeography

The tropical lowland forests of the Magdalena River Valley, located in northern Colombia, encompass two dissimilar biogeographic units: the Chocó-Tumbes-Magdalena and the Tropical Andes (Myers et al. 2000). The Central and Eastern Cordillera bound this biologically rich Valley, which is characterized by high levels of endemism (Gentry 1992). The unique biological composition of this region is partly explained by its geographic isolation and the presence of the high-elevation mountain ranges that act as geographic barriers. We documented the presence of a number of euglossine bee taxa that were previously known to occur only in Central America and the Choco region of Colombia, such as *Euglossa championi* Cheesman, *E. dressleri* Moure*, E. hansoni* Moure*,* and *E. gorgonensis* Cheesman. At the same time, we also recorded species that were previously known only from the Amazon region, such as *Aglae caerulae*, *Euglossa orellana,* and *E. intersecta*. This observation highlights the importance of this region for biogeographic studies and conservation purposes. The lowland inter-Andean forests of Colombia are one of the most threatened ecosystems in the neotropical region due to pervasive habitat destruction, fragmentation, large-scale cattle ranching, agriculture, and illegal mining. Recent studies have estimated that in 2010 almost 78% of the original land cover of lowland forests in the Magdalena River Valley had been lost (Etter et al. 2006; Armenteras et al. 2010), and most of the remaining forest fragments face imminent threats such as large-scale oil palm plantations (Link et al. 2013).

## Supporting Information

**Table S1.** List of species and their abundance at the three sampled sites: Reserva, Guinea and Rio Claro. **Table S2.** List of the six dominant species for each of the three sampled sites and totals. **Table S3.** Diversity index for the three sampled sites. **Table S4.** Similarity coefficients of pairwise-site comparisons. **Table S5.** Summary table of studies on euglossine bee communities. **Figure S1.** Heatmap showing clustering of euglossine bee communities as they were sampled monthly. **Figure S2.** Rarefaction curves of species accumulation in Reserva, Guinea, Rio Claro, and all sites combined. **Figure S3.** Species-rank curves for three communities of euglossines bees, and all communities combined. A lognormal function was fitted to each distribution. **Figure S4.** Total number of species (A) and individuals (B) attracted to the five chemical bait used for sampling: Cineol (Cin), Dimethoxybenzene (DMB), Methyl Cinnamate (MC), Methyl Salycilate (MS) and Vainillina (VAI). **Figure S5.** Standardized Effect Size Mean Pairwise Phylogenetic Distance (SESMPD) for three euglossine bee communities over the course of a year. Dashed line indicates the relative rainfall pattern fluctuations. **Figure S6.** Functional Diversity of three euglossine bee communities over the course of a year. Dashed line indicates the relative rainfall pattern fluctuations. **Figure S7.** Functional Richness of three euglossine bee communities over the course of a year. Dashed line indicates the relative rainfall pattern fluctuations. **Figure S8.** Functional Divergence of three euglossine bee communities over the course of a year. Dashed line indicates the relative rainfall pattern fluctuations. **Figure S9.** Functional Evenness of three euglossine bee communities over the course of a year. Dashed line indicates the relative rainfall pattern fluctuations. **Figure S10.** Phylogenetic community structure of sampled communities in Rio Claro site. **Figure S11.** Phylogenetic community structure of sampled communities in Reserva. **Figure S12.** Phylogenetic community structure of sampled communities in Guinea.

**Data S1.** DNA alignment used for phylogenetic analysis.
