# Supplementary material for: Seasonal cycles, phylogenetic assembly, and functional diversity of orchid bee communities
Source: Ecol Evol. 2015 Apr 13;5(9):1896–907. doi: 10.1002/ece3.1466 (PMC4485970; doi:10.1002/ece3.1466)

Supplementary Table 1. List of species and their abundance at the three sampled sites: Reserva, Guinea and Rio Claro.

	Reserva	Guinea	Rio Claro
Aglae cerulea	0	1	0
Eufriesea concava	1	1	0
Eufriesea lucifera	2	0	0
Eufriesea ornata	0	3	0
Eufriesea pulchra	19	19	1
Euglossa allosticta	10	1	0
Euglossa amazonica	37	30	63
Euglossa bursigera	47	42	65
Euglossa championi	0	5	2
Euglossa chlorina	164	161	2
Euglossa cognata	181	195	3
Euglossa cordata group 4	35	35	1
Euglossa cordata group 6	7	7	12
Euglossa cordata group 7	3	3	0
Euglossa cordata group 8	9	23	5
Euglossa cordata group 9	1	0	0
Euglossa crassipunctata	65	27	21
Euglossa cybelia	5	35	0
Euglossa deceptrix	0	0	3
Euglossa despecta	108	24	44
Euglossa dissimula	3	7	2
Euglossa dodsoni	0	0	1
Euglossa dressleri	6	38	3
Euglossa flammea	9	4	0
Euglossa gorgonensis	4	49	56
Euglossa hansoni	0	0	2
Euglossa heterosticta	30	26	4
Euglossa ignita	313	188	161
Euglossa igniventris	0	0	3
Euglossa imperialis	337	169	422
Euglossa intersecta	18	6	0
Euglossa ioprosopa	0	2	0
Euglossa laevicincta	0	1	0
Euglossa liopoda	57	18	27
Euglossa maculilabris	4	0	2
Euglossa mixta	161	10	59
Euglossa orellana	78	59	36
Euglossa platymera	0	206	3
Euglossa s st.	1	4	2
Euglossa sp1	3	46	0
Euglossa tridentata	18	18	68
Euglossa turbinifex	2	0	0
Euglossa variabilis	35	19	5
Eulaema boliviensis	1	0	0
Eulaema bombiformis	5	5	1
Eulaema cingulata	155	63	17
Eulaema meriana	47	58	6
Eulaema nigrita	3	0	0
Exaerete frontalis	27	11	31
Exaerete smaragdina	2	0	19

Supplementary Table 2. List of the six dominant species for each of the three sampled sites and totals. Number of individuals per species (n), percentage (%), and rank within each category.

Species	Total			Reserva			Guinea			Rio Claro		
	n	%	Rank	n	%	Rank	n	%	Rank	n	%	Rank
Euglossa imperialis	928	19.3	1	337	16.7	1	169	10.4	3	422	36.6	1
Euglossa ignita	662	13.8	2	313	15.5	2	188	11.6	2	161	13.9	2
Euglossa cognata	379	7.9	3	181	8.9	3	195	12	1	3	0.2	20
Euglossa chlorina	327	6.8	4	164	8.14	4	161	9.9	4	2	0.17	21
Euglossa cingulata	235	4.9	5	155	7.6	5	63	3.8	5	17	1.47	14

Supplementary Table 3. Diversity index for the three sampled sites. Observed number of species (S), estimated species richness through Jacknife (N(JK)), standard error of Jacknife estimator (SE), Shanon-Wiener diversity index (H'), Evenness (E), Simpson diversity index (D) and rarefaction index (R).

	**S**	**N(JK)**	**SE**	**H'**	**E**	**D**	**1-D**	**1/D**	**R**
**Reserva**	37	39	2	2.76	0.77	0.09	0.91	11.63	43.32
**Guinea**	40	43	2.45	3.00	0.81	0.07	0.93	14.65	47.13
**Rio Claro**	34	38	2.82	2.38	0.67	0.17	0.83	5.94	49.00

Supplementary Table 4. Similarity coefficients of pairwise-site comparisons. Shared number of species, Jaccard coefficients, Sorensen coefficients, estimator of Jaccard coefficient using Chao's correction, estimator of Sorensen coefficient using Chao's correction, Morisita-Horn coefficient, Bray-Curtis coefficient, and β-diversity.

	**Shared Species**	**Jaccard**	**Sorensen**	**Chao-Jaccard**	**Chao-Sorensen**	**Morisita-Horn**	**Bray-Curtis**	**β diversity**
**Reserva x Guinea**	34	0.791	0.883	0.982	0.991	0.924	0.725	0.018
**Reserva X Rio Claro**	26	0.578	0.732	0.981	0.99	0.705	0.529	0.019
**Guinea X Rio Claro**	27	0.574	0.73	0.938	0.968	0.574	0.52	0.062

Supplementary Table 5. Summary table of studies on euglossine bee communities. Reference of the study, locality sampled, number of individuals (N), richness (S), Shanon index (H'), Simpson's index (D), dominant species, number of baits, method of collection, number of days sampled and type of habitat.

Study	Locality	N	S	H'	1/D	Dominant sp.	Baits	Method	Length	Habitat
(Dodson et al. 1969)	Panama, Canal	927	48				5	net	5 days	
**Braga 1976~~??~~**	**Brazil, Amazonas**	**160**	**42**				**5**	**net**		
(Janzen et al 1982)	Costa Rica, Corcovado	961	27	2.37	0.34	*Eg. imperialis*	5	net	6 days	rain forests
(Janzen et al 1982)	Costa Rica, Santa Rosa	1200	21	1.28	0.70	*Eg. viridissima*	5	net	4 days	deciduous forest
(Ackerman 1983)	Panama, BCI	21842	44	2.43	0.26	*Eg. tridentata*	16	net	57 days	seasonally dry forest
(Pearson & Dressler 1985)	Peru, Tambopata	2917	39			*Eg. ignita*	5	net	23 days	rain forest
(Powell & Powell 1987)	Brazil, Manaus	992	16			*Eg. chalybeata*	3	net*	8 days	
(Roubik & Ackerman 1987)	Panama, **3 sites**					*Eg. imperialis*				wet, moist, cloud for
(Wittmann et al 1988)	Rio Grande do Sul, BR	639	5	0.13		*Ef. violacea*		net		
(Ackerman 1989)	BCI, PA	27874	53							
(Rebêlo & Garófalo 1991)	São Paulo, BR	892	8	1.33		*Eg. pleosticta*				
(Becker et al 1991)	Amazonas, BR	290	16	1.36		*Eg. stilbonata*				
Morato et al. 1992	Amazonas, BR	1242	27	2.21		*Eg. stilbonata*				
Morato 1994	Amazonas, BR	838	25	2.27		*Eg. stilbonata*				
Oliveira & Campos 1995	Amazonas, BR	2422	38	2.34		*Eg. stilbonata*				
Rebêlo & Garófalo 1997	Bahia, BR	1144	12	0.97		*El. nigrita*				
Rebêlo & Cabral 1997	Maranhão, BR	1153	9	1.18		*Eg. cordata*				
Neves & Viana 1999	Bahia, BR	527	7	1.13						
Silva & Rebelo 1999	Maranhão, BR		37	2.57			5			

Brito & Rêgo	Maranhão, BR	467	19	2.11		*Eg. piliventris*				
Tonhasca et al 2002	Rio Janeiro, BR	3653	21	2.00		*El. nigrita*				
Nemesio 2003	Minas Gerais, BR	122	7	1.48		*El. cingulata*				
Otero & Sandino 2003	Choco, COL	2008	31	2.25		***El. cingulata***		net	22 mon	Farm, 2nd and old
Nemésio & Morato 2004	Acre, BR	254	22	2.01		*El. cingulata*				
Sofia et al. 2004	Paraíba, BR	434	9	1.37		*Ef. violacea*				
Sofia & Suzuki 2004	Paraíba, BR	245	7	1.52		*Ef. violacea*				
Marins & Souza 2005	Paraíba, BR	1151	11	0.75		*El. nigrita*				
Milet-Pinheiro & Schlindwein 2005	Pernambuco, BR	945	16	1.20		*Eg. cordata*				
Nemésio 2005	Roraima, Br	90	12	2.16		*Ef. superba*				
Souza et al. 2005	Paraíba, BR	2314	11	0.81		*Eg. cordata*	6			Forest/savanna
Nemésio & Morato 2006	Acre, Brazil	1744	33			*El. cingulata*	7	net/traps		forested area
Nemesio & Silveira 2006	Minas Gerais, BR	918	18	1.67		*Eg. analis*				
Farias et al 2008	Paraíba, BR		7	0.22						duna/restinga
Nemésio 2008	Minas Gerais, BR	234	9			*Eg. truncata*	5		1 year	Alt. grad forest frag
Storck-Tonon et al 2009	Acre, BR	3675	36	2.54		*El. cingulata*	6	net/trap	48 days	
Rasmussen 2009	Loreto, Peru	2072	33	2.314	5.045	*Eg. ignita*	5	net	14 mth	lowland rainforest
Silva et al 2009	Maranhão, BR	429	14	1.93		*El. cingulata*	5	net/trap		restinga
Brosi 2009	Las Cruces, CRI		23			*Eg. championi*		trap		
**This study**	**Colombia, Quinchas**	**5666**	**49**	**3.00?**		***Eg. imperialis***	**5**	**net**	**11 mth**	

Supplementary Figure 1. Heatmap showing clustering of euglossine bee communities as were sampled monthly.

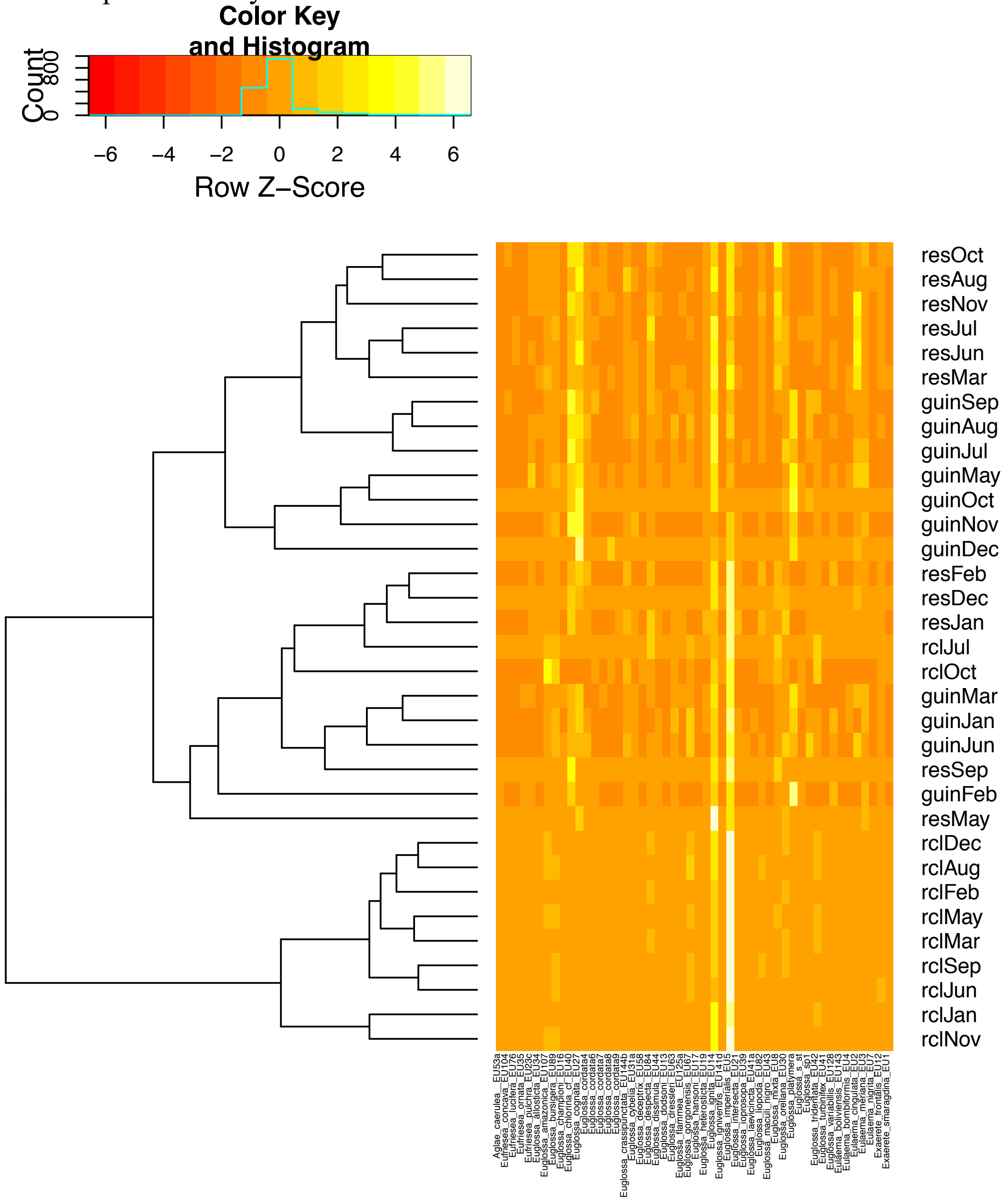

Supplementary Figure 2. Rarefaction curves of species accumulation in Reserva, Guinea, Rio Claro, and all sites combined.

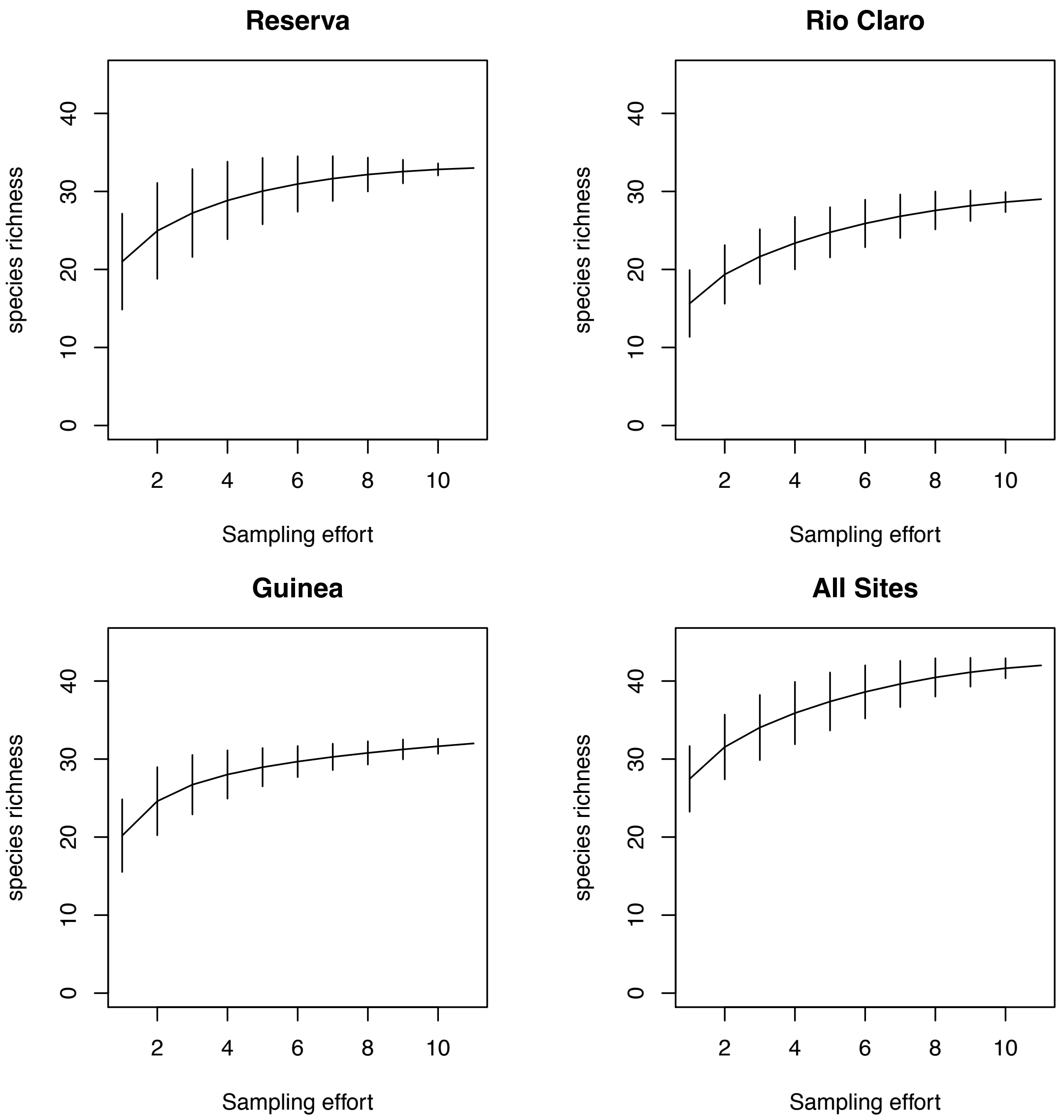

Supplementary Figure 3. Species-rank curves for three communities of euglossines bees, and all communities combined. A lognormal function was fitted to each distribution.

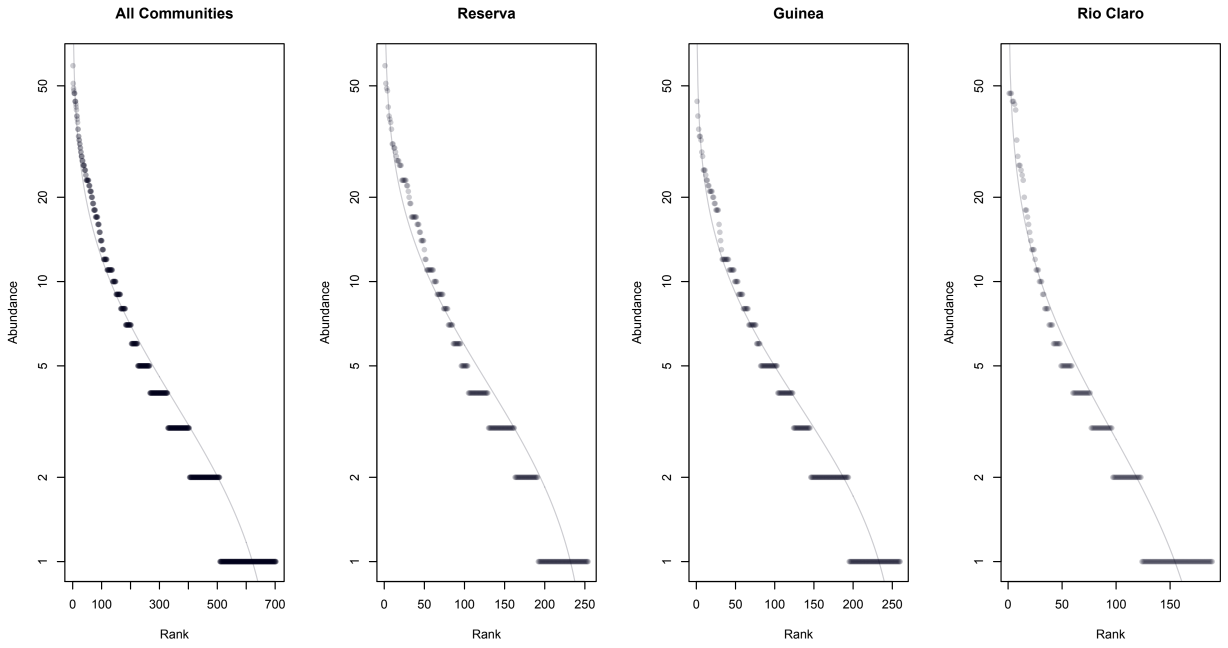

Supplementary Figure 4. Total number of species (A) and individuals (B) attracted to the five chemical bait used for sampling: Cineol (Cin), Dimethoxybenzene (DMB), Methyl Cinnamate (MC), Methyl Salycilate (MS) and Vainillina (VAI).

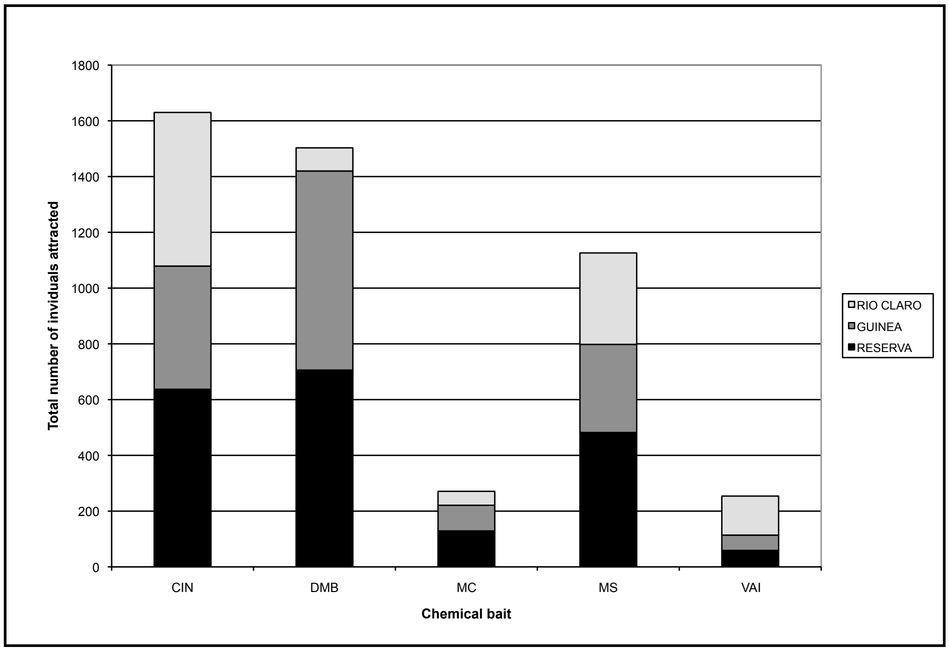

Supplementary Figure 5. Standardized Effect Size Mean Pairwise Phylogenetic Distance (SES-MPD) for three euglossine bee communities over the course of a year. Dashed line indicates the relative rainfall pattern fluctuations.

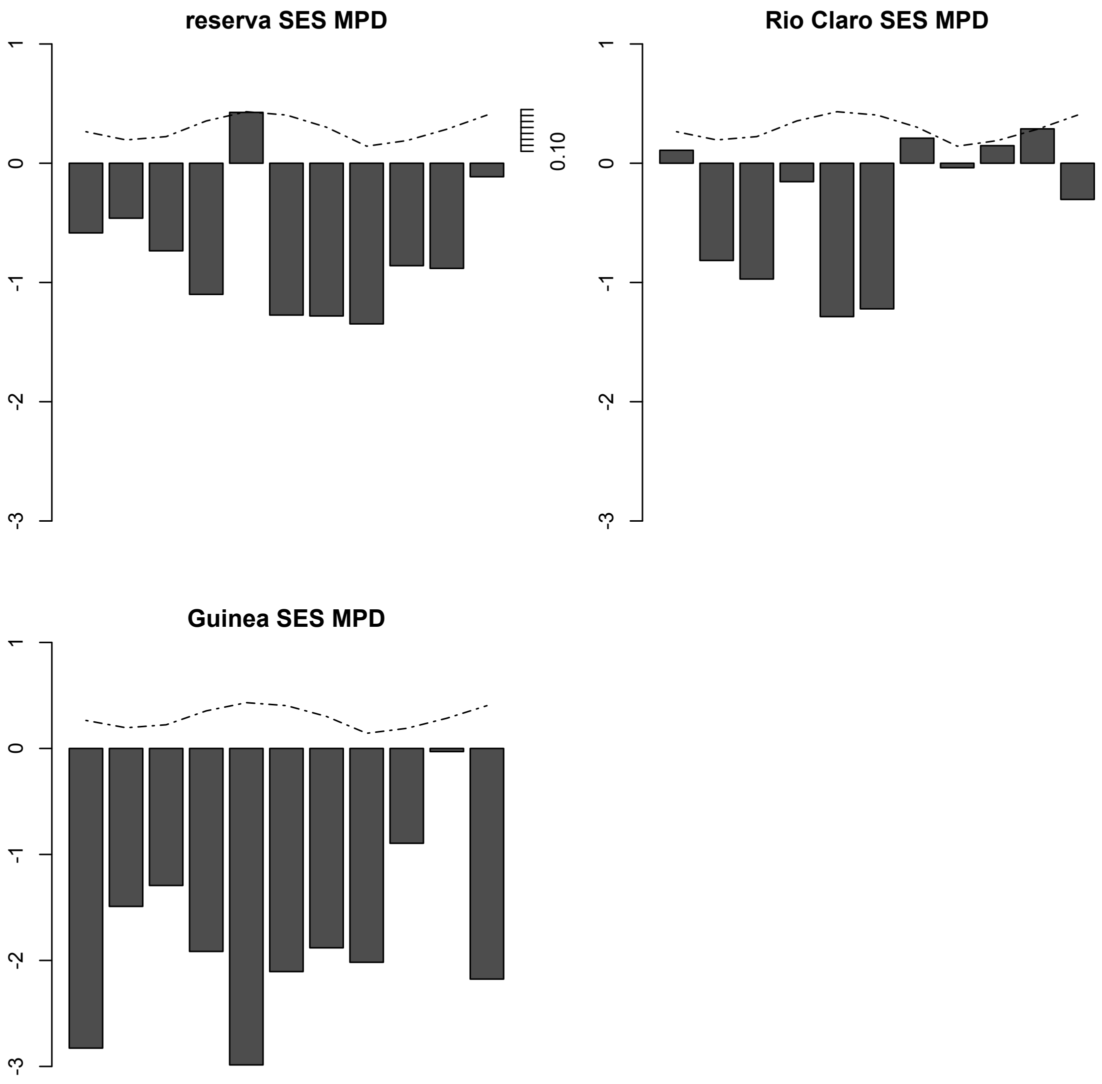

Supplementary Figure 6. Functional Diversity of three euglossine bee communities over the course of a year. Dashed line indicates the relative rainfall pattern fluctuations.

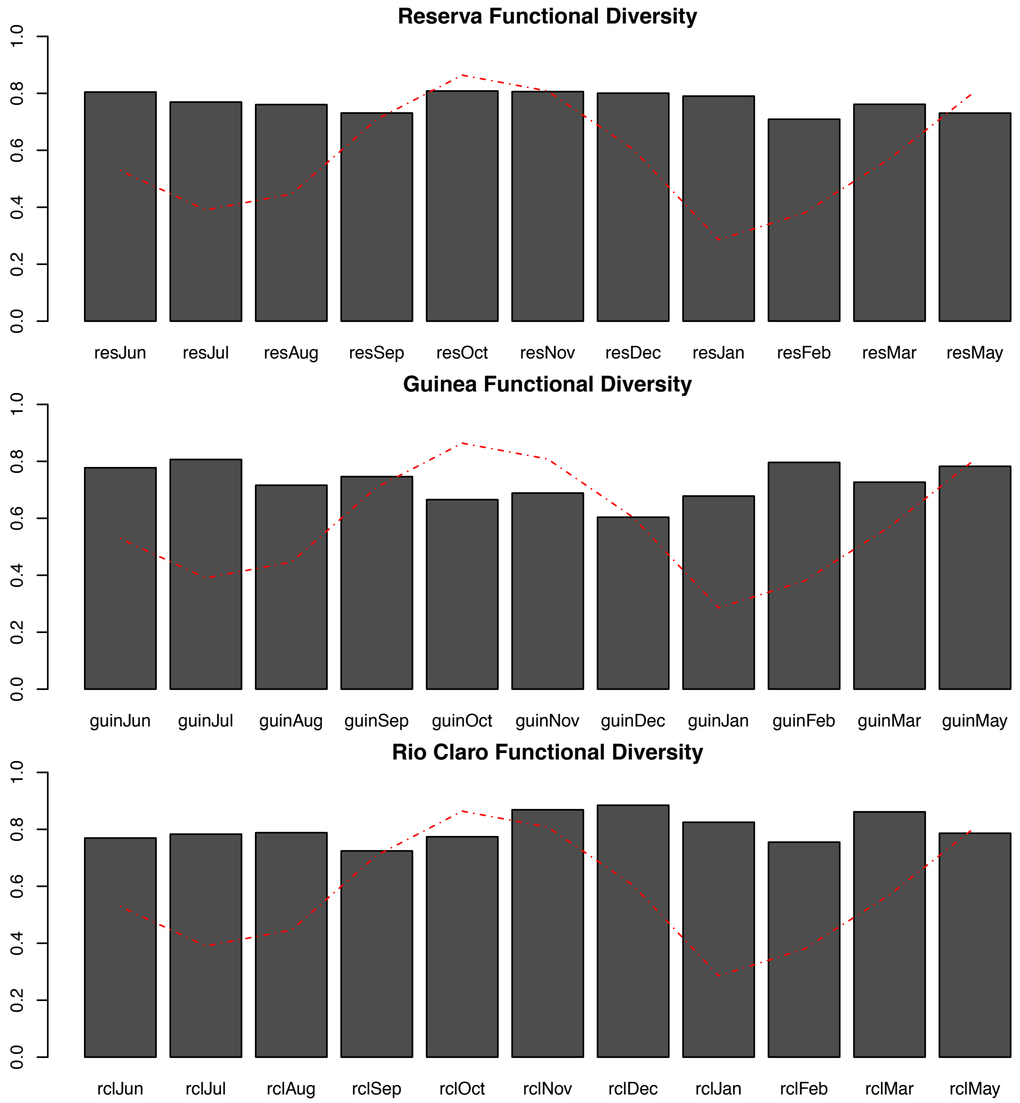

Supplementary Figure 7. Functional Richness of three euglossine bee communities over the course of a year. Dashed line indicates the relative rainfall pattern fluctuations.

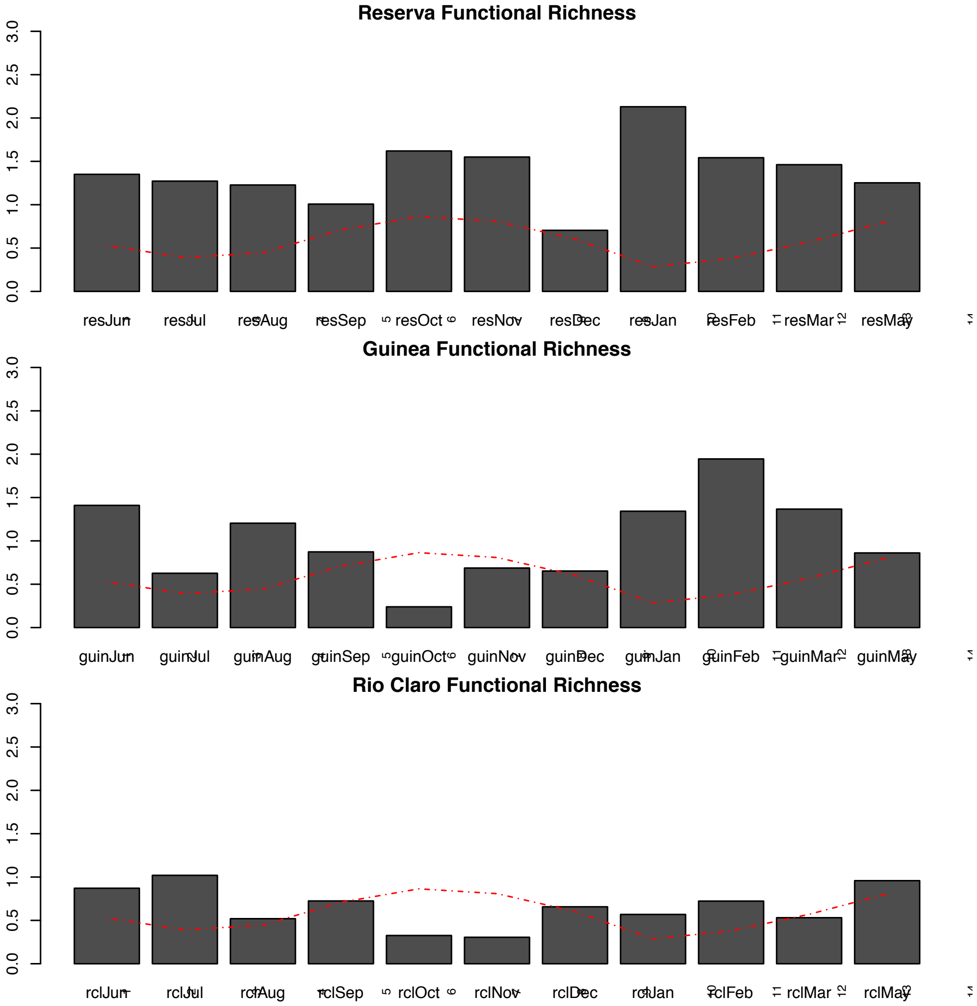

Supplementary Figure 8. Functional Divergence of three euglossine bee communities over the course of a year. Dashed line indicates the relative rainfall pattern fluctuations.

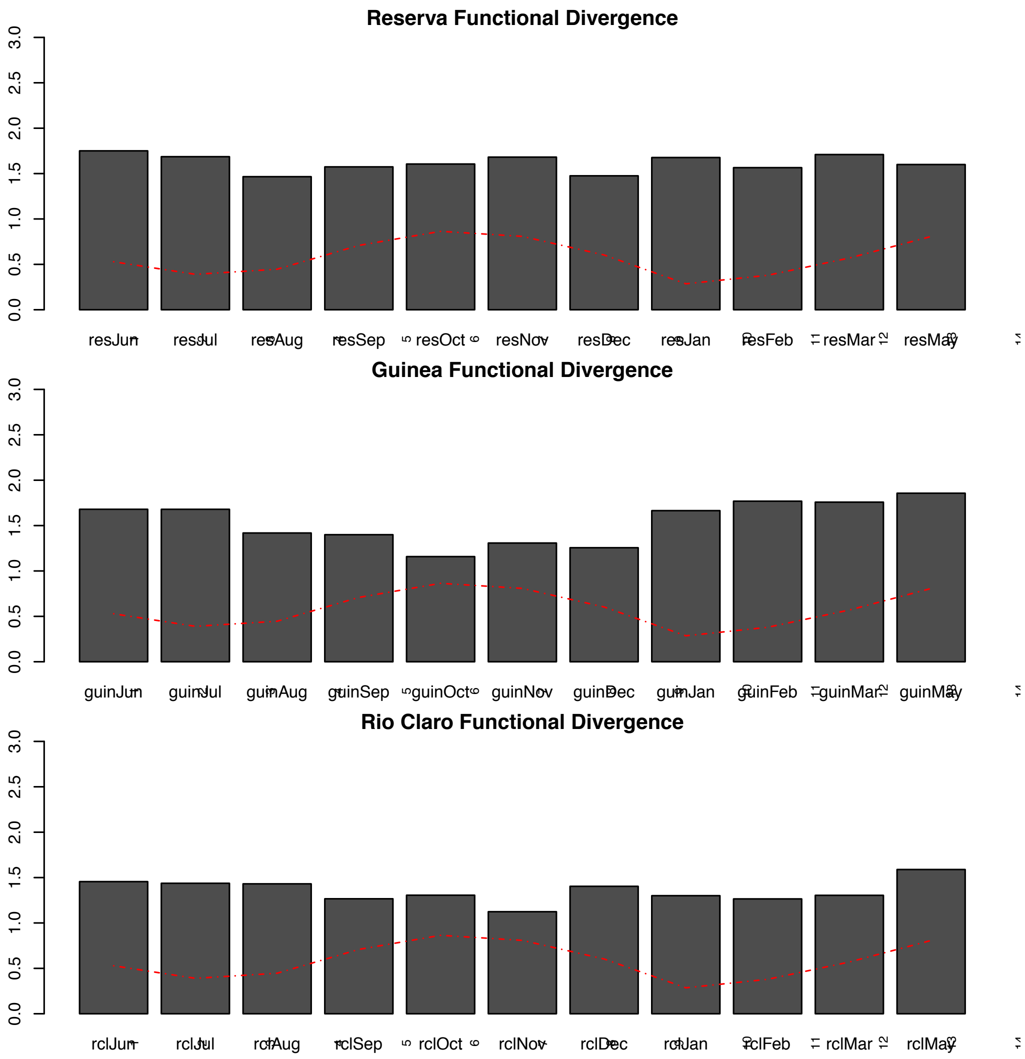

Supplementary Figure 9. Functional Evenness of three euglossine bee communities over the course of a year. Dashed line indicates the relative rainfall pattern fluctuations.

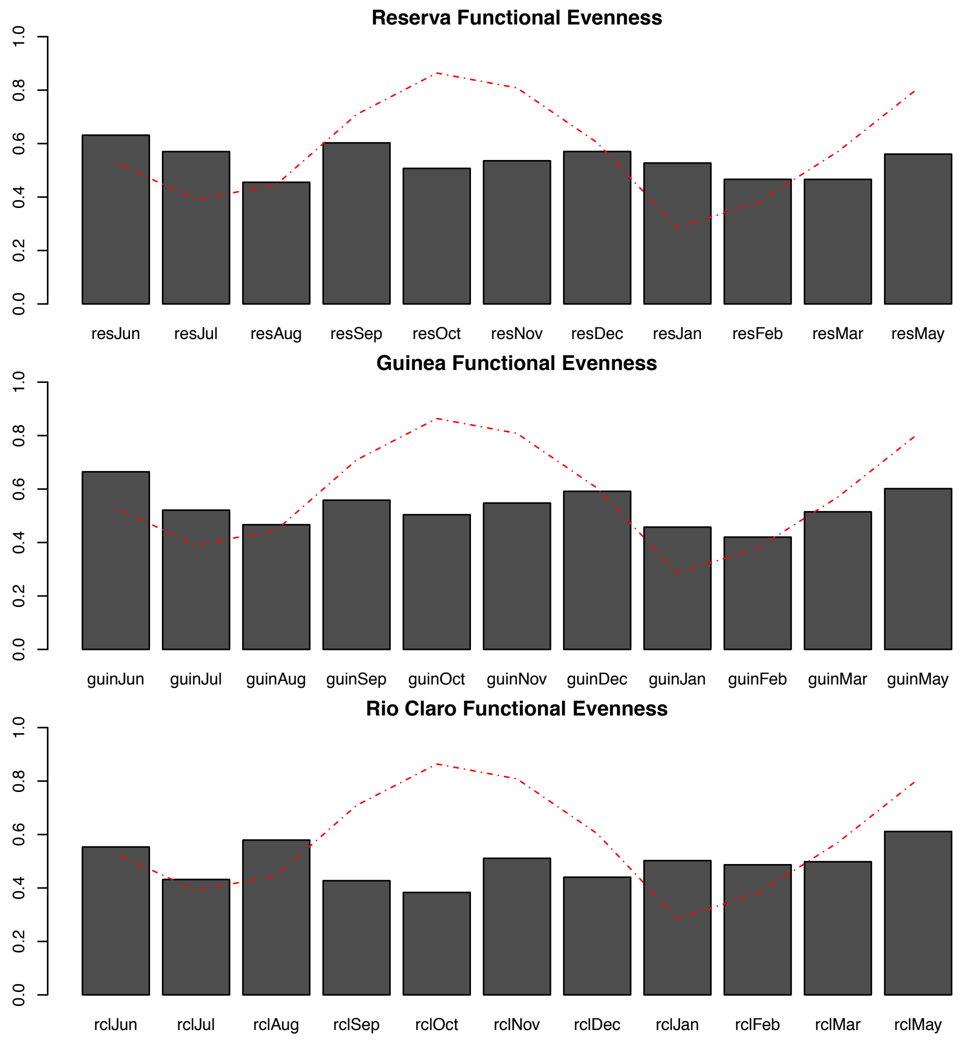

Supplementary Figure 10. Phylogenetic community structure of sampled communities in the Rio Claro site.

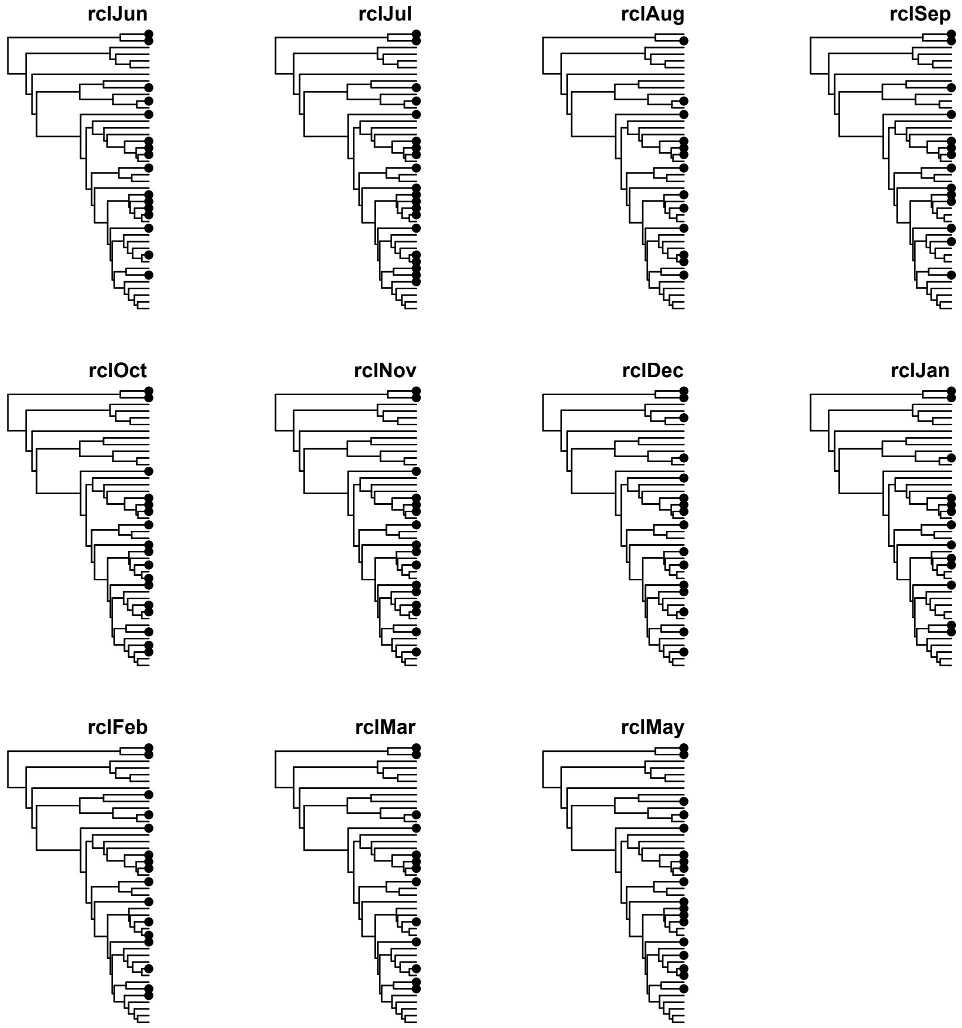

Supplementary Figure 11. Phylogenetic community structure of sampled communities in the Reserva.

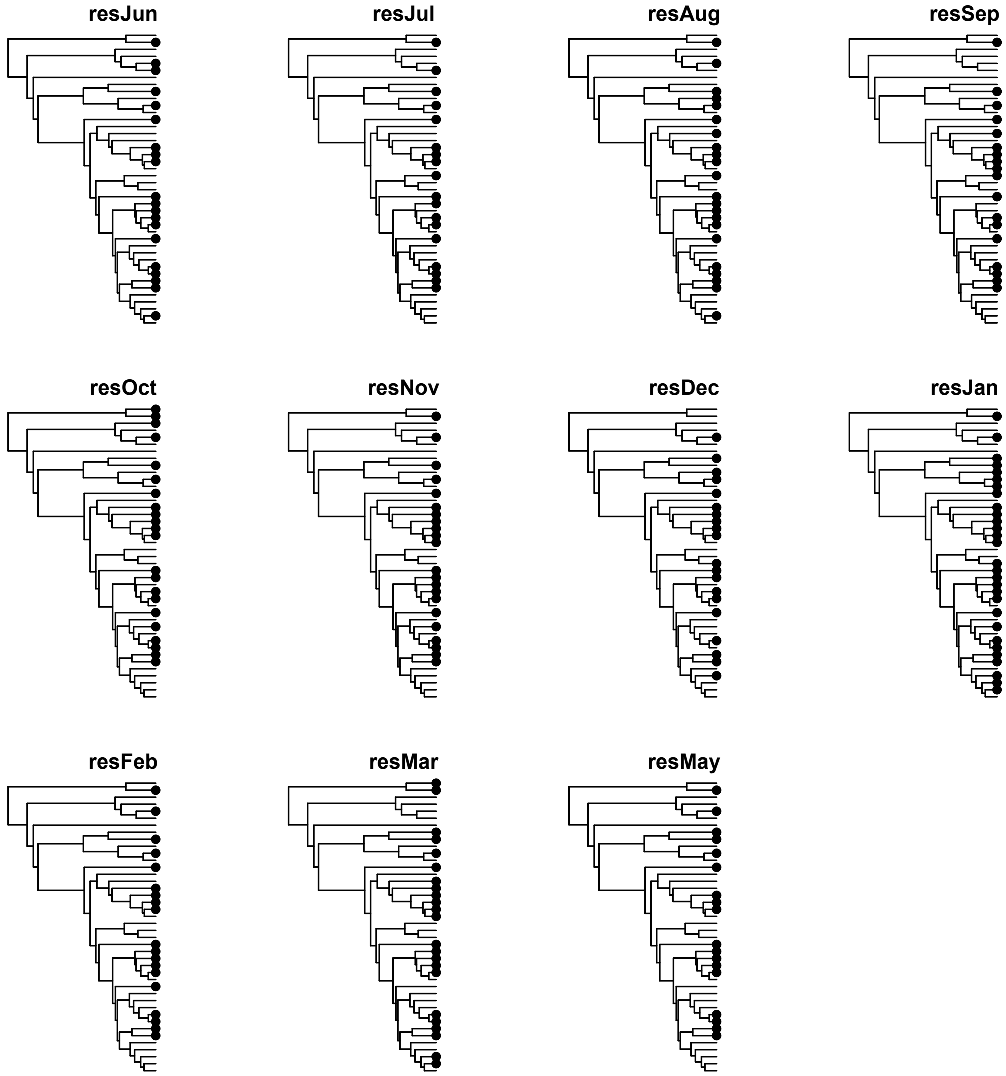

Supplementary Figure 12. Phylogenetic community structure of sampled communities in the Guinea.

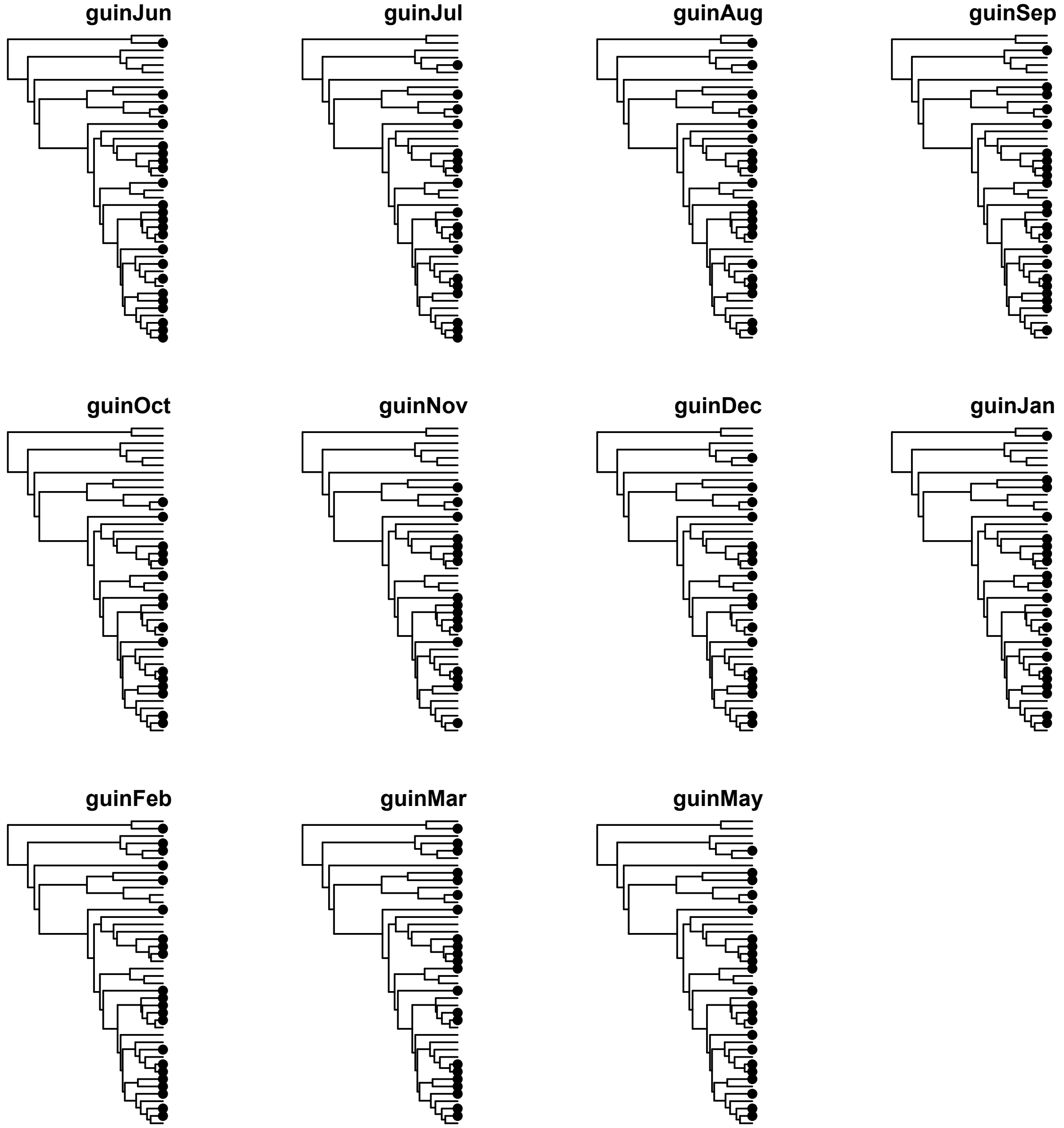

Supplement: Supplementary file 1 [file ece30005-1896-sd1.pdf]
